# Investigating suicidality across the autistic-catatonic continuum in a clinical sample of subjects with major depressive disorder and borderline personality disorder

**DOI:** 10.3389/fpsyt.2023.1124241

**Published:** 2023-05-18

**Authors:** Liliana Dell’Osso, Benedetta Nardi, Chiara Bonelli, Davide Gravina, Francesca Benedetti, Giulia Amatori, Simone Battaglini, Gabriele Massimetti, Mario Luciano, Isabella Berardelli, Natascia Brondino, Marianna De Gregorio, Giacomo Deste, Marta Nola, Antonino Reitano, Maria Rosaria Anna Muscatello, Maurizio Pompili, Pierluigi Politi, Antonio Vita, Claudia Carmassi, Ivan Mirko Cremone, Barbara Carpita, Mario Maj

**Affiliations:** ^1^Department of Clinical and Experimental Medicine, University of Pisa, Pisa, Italy; ^2^Department of Psychiatry, University of Campania “Luigi Vanvitelli”, Naples, Italy; ^3^Department of Neuroscience, Mental Health and Sense Organs, University of Roma “La Sapienza”, Rome, Italy; ^4^Department of Brain and Behavioral Sciences, University of Pavia, Pavia, Italy; ^5^Department of Biomedical and Dental Sciences and Morphofunctional Imaging, University of Messina, Messina, Italy; ^6^Department of Clinical and Experimental Sciences, University of Brescia, Brescia, Italy

**Keywords:** catatonia spectrum, mood disorder, major depression disorder (MDD), borderline personality disorder (BPD), suicide, catatonic traits, catatonia, autistic-catatonic continuum

## Abstract

**Background:**

Recent literature has highlighted that catatonia may be more prevalent among psychiatric patients than previously thought, beginning from autism spectrum disorders (ASD), for which it has been suggested to represent a severe late consequence, but also among individuals with mood disorders and borderline personality disorder (BPD). Interestingly, one critical point shared by these conditions is the increased risk of suicidality. The aim of this study was to evaluate how the presence and the prevalence of catatonic symptoms may shape and correlate with suicidal risk in a sample of individuals with major depressive disorder (MDD) or BPD.

**Methods:**

We recruited two clinical samples of subjects (BPD and MDD) and a control group without a diagnosis according to DSM-5 (CTL). Subjects were assessed with the catatonia spectrum (CS) and the MOODS-SR for evaluating suicidality.

**Results:**

In the total sample, suicidality score was significantly and positively correlated with all CS domains and CS total score. Correlation and regression analyses highlighted specific patterns of association among Catatonia spectrum domains and suicidality in the MDD and BPD group and in the total sample.

**Conclusion:**

In both disorders, higher catatonic traits are linked to higher suicidal tendencies, confirming the high risk of suicide for this population. However, different patterns of association between catatonic symptoms and suicidality were highlighted in the two disorders.

## 1. Introduction

The term “catatonia” was firstly introduced by Kahlbaum in 1874 (1) referring to a composite picture of behavioral and motor disturbances (such as negativism, mutism and automatisms) along with cognitive, affective and neurovegetative manifestations. Interestingly, Kahlbaum observed a high frequency of acute alterations of mood in catatonic subjects, of both polarities, underlined the similarity with manic-depressive psychosis (1). Today, the DSM-5-TR recognizes catatonia as a neuropsychiatric condition included in the chapter “Spectrum of Schizophrenia and Other Psychotic Disorders,” and characterized by the occurrence of three or more of 12 symptoms (stupor, catalepsy, waxy flexibility, negativism, fixed posture, mannerisms, stereotypes, agitation, presence of grimacing, echolalia, and echopraxia) (2). The DSM-5-TR features three main categories of catatonia: “catatonia associated with another mental disorder,” “catatonic disorder due to another medical condition,” and “catatonia without specification.” Even though the current categorization of catatonia still follows Bleuler conceptualization (3), over the years many authors argued that it should be recognized as a separate syndrome (4–6). Noticeably, catatonic symptoms may occur with a multitude of medical and organic brain conditions (7). Recent literature also highlighted that catatonia may be more prevalent among psychiatric patients than previously thought, in particular among younger patients (8, 9), patients with autism spectrum disorder (ASD) (10–14) and individuals with mood disorders rather than with non-affective disorders like schizophrenia (15); in particular, catatonic signs have been reported to be associated with mood disorders in 13% to 31% cases (16–18). One of the most recent studies reported also increased levels of catatonic symptoms in a sample of borderline personality disorder (BPD) patients, even higher that those found in patients with major depression disorder (MDD) (19). Interestingly, one of the most critical points shared by mood disorders and BPD is the increased risk of suicidality. On the other hand, the literature also reported a higher suicidal risk linked to catatonia, especially when of psychiatric origin (20, 21).

MDD with chronic course and recurrent episodes is a major affective disorder with a chronic course characterized by recurrent depressive episodes (2) known to be associated with a high risk for suicidality (22, 23). Catatonic symptoms have already been described in depressive state (24, 25) and in other mood disorders, especially with mixed states. In this framework two studies, reported a history of an index episode with mixed features in 61% and 73% catatonic patients, respectively (26, 27). Interestingly, patients with mixed states also typically show greater suicidal behavior and are at higher risk of suicide (28).

Another disorder with a well-known association with higher levels of self-injurious and suicidal behaviors is BPD (29, 30). BPD is characterized by a pervasive pattern of instability in different areas, in particular regarding emotional regulation, interpersonal relationships, self-image, and control of impulses (31). BPD is therefore an extremely relevant disorder associated with severe functional impairment and high rates of suicide, affecting almost 20% and 10% of psychiatric in- and out-patients, respectively (29, 30). Despite seeming quite different, the recent literature increasingly reported significant overlaps between BPD and ASD. For instance, although being characteristics of BPD, strong relationships and superficial friendships, as well as the propensity to acting out instead of verbalizing emotions, are also prevalent in ASD (32). In a similar way, while essential characteristics of ASD, impairments in verbal and nonverbal communication, social functioning, incorrect assumptions about intentions, and emotional outbursts are also frequently documented in BPD subjects (33–40). Other traits shared by BPD and ASD are a high proportion of self-harming behaviors (32), pervasive patterns of emotional dysregulation (41, 42) and difficulties in the Theory of Mind (43). On the other hand, to date, the pathophysiology of BPD is still relatively unexplored, and its correlation with ASD is based mainly on the shared clinical and psychological features. However, evidences from some researches may suggest that ASD and BDP may share similar neurobiological alteration such as an atypical functionality of the hypothalamus-pituitary-adrenal (HPA) axis that has been widely reported in both disorders (44–46) and an alteration of the endocannabinoid and oxytocin system, which are important for the emotional regulation and social interaction (47, 48).

In this framework, it is interesting to acknowledge that catatonic symptoms and autistic traits have been found to be particularly prevalent on the BPD population (19, 49, 50). Moreover, previous studies already reported how in both BPD and catatonia a higher prevalence of autistic traits would be associated with a greater severity of the manifestations and an increased suicidal risk (51–53).

Even if scientific literature reports overlapping features between mood disorders and BPD, in terms of mood deregulation, affective instability, impulsivity, suicidal ideation and behaviors (54, 55), they remain very distinct and different clinical conditions, not only from a psychopathological point of view, but also in terms of illness course, gender prevalence and heritability features.

In this framework the aim of this study was to evaluate how the presence and the prevalence of catatonic symptoms may shape and correlate with suicidal risk in a sample of individuals with MDD or BPD.

## 2. Materials and methods

A consecutive sample of subjects with a clinical diagnosis of MDD, BPD or catatonia were recruited in six Italian University Departments of Psychiatry, coordinated by the University of Pisa: University of Campania “Luigi Vanvitelli,” University of Pavia, University of Messina, La Sapienza University of Rome, University of Catania, and University of Brescia. Exclusion criteria for all groups were: age under 18 years, language or intellectual impairment affecting the possibility to fulfill the assessments, mental disability, poor cooperation skills, and ongoing psychotic symptoms. The study was conducted in accordance with the Declaration of Helsinki. The Ethics Committee of the Azienda Ospedaliero-Universitaria of Pisa approved all recruitment and assessment procedures. Written informed consent was obtained from all participants after they received a complete description of the study and had the opportunity to ask questions. Subjects were not paid for their participation according to Italian legislation.

The Structured Clinical Interview for DSM-5, Research Version (SCID-5-RV) (56) was used to confirm the diagnosis of BPD and MDD, as well as the absence of mental disorders among HC subjects. All subjects were assessed with the following self-report instruments: the catatonia Spectrum (CS) and the mood spectrum self-report (MOODS-SR). The questionnaires were compiled autonomously by all the subjects.

### 2.1. The catatonia spectrum

The CS is a self-assessment questionnaire that investigates nuclear, subthreshold, atypical and partial manifestations of catatonia across the lifespan, developed and validated at University of Pisa (19). It consists of 74 items divided into 8 domains investigating the main criteria of the catatonia diagnosis: (1) *psychomotor activity* (stupor); (2) *verbal response* (mutism); (3) *repetitive movements* (stereotypes); (4) *artificial expressions and actions* (mannerisms); (5) *oppositivity or poor response to stimuli* (negativism); (6) *response to instructions given from outside* (automatic obedience); (7) *automatisms*; (8) *impulsivity*. For each item there is a dichotomous answer “yes” and “no.”

The CS showed great validity and reliability, excellent internal consistency and test–retest reliability and a significant and positive convergent validity with alternative dimensional measures of catatonia (19).

### 2.2. The MOODS-SR

The MOODS-SR is a questionnaire tailored to evaluate the presence of a broad range of mood symptoms and temperamental traits during lifetime. It consists of 160 items and is divided in 7 domains. For each item there is a dichotomous answer “yes” and “no.” For the Italian version the Cronbach’s alphas of the instrument ranged between 0.72 and 0.92. The MOODS-SR was employed in previous studies for evaluating suicidality (suicidal ideation and behaviors) (51, 57), as measured by items 102 to 107 of the instrument.

### 2.3. Statistical analysis

ANOVA and chi-square analyses were performed in order to compare among group mean MOODS suicidality scores and the presence of at least one positive answer to MOODS suicidality items. Pearson’s correlation coefficient was used for evaluating the pattern of correlations among the scores reported on psychometric instruments within the BPD, MDD and CTL subjects. Subsequently, in order to evaluate which CS domains were statistically predictive of suicidality score in the sample, linear regression analyses were performed with suicidality score as the dependent variable and CS total and domain scores as independent variables. Moreover, logistic regression analyses were performed with the presence of a positive answer to at least one suicidality item of MOODS suicidal ideation and MOODS suicidal behavior as the dependent variables and CS total and domain scores as independent variables. All statistical analyses were conducted using Statistical Package for the Social Sciences (SPSS) version 26.0.

## 3. Results

The sample was composed of 147 MDD subjects, 105 subjects with BPD and 156 CTL. Sociodemographic data of the sample are reported elsewhere (58).

Considering the presence of positive answers to at least one MOODS suicidality item and one MOODS suicidal thoughts item, the CTL group reported a percentage significantly lower (17.9%; 17.3%) compared to the BPD (76.4%; 76.4%) and the MDD domain (65.3%; 63.9%), while no significant differences were reported in the two pathological groups. On the other hand, when evaluating the presence of at least one positive answer to MOODS suicidal behavior items, the CTL group reported a significantly lower percentage (3.2%) compared to the MDD group (37.4%), and both groups showed a significantly lower percentage compared to the BPD group (60.4%) (see Table 1).

**Table 1:** Presence or absence of a positive answer to at least one item of MOODS suicidality, suicidal ideation and behavior, sorted by diagnosis.

		CTL (*N* = 156)	MDD (*N* = 147)	BPD (*N* = 105)	*χ* ^2^
		*N* (%)	*N* (%)	*N* (%)	
MOODS suicidality	Absence	128 (62.7)	51 (34.7)	25 (23.6)	107.461
	Presence	28 (17.9)	96 (65.3)	81 (76.4)	
Suicidality ideation	Absence	129 (82.7%)	53 (36.1)	25 (23.6)	107.668
	Presence	27 (17.3)	94 (63.9)	81 (76.4)	
Suicidal behavior	Absence	151 (96.8)	92 (62.6)	42 (39.6)	103.123
	Presence	5 (3.2)	55 (37.4)	64 (60.4)	

^*^p < 0.05; presence: MDD, BPD > CTL; ^#^*p* < 0.05; presence: BPD > MDD > CTL.

In the total sample, as well as in the MDD group, the total suicidality score, the suicidal ideation and the suicidal behaviors scores, were significantly and positively correlated with all CS domains and CS total score (see Tables 2 3). In the BPD group, the suicidality total score and suicidal ideation score were significantly and positively correlated with CS total score and all CS domains except for *artificial expressions*, while suicidal behavior score was significantly and positively correlated with CS total score and all CS domains except for *artificial expressions* and *repetitive movements* (Table 4). Finally, in the CTL group, the total suicidality score and the suicidal ideation were significantly and positively correlated with all CS domains and CS total score, while suicidal behaviors scores, were significantly and positively correlated with CS total score and all CS domains except for *verbal response*, *artificial expressions* and *repetitive movements* (Table 5).

**Table 2:** Pearson’s correlations coefficients (*r*) among MOODS suicidality score and CS scores in the total sample.

CS scores	Suicidality	Suicidality ideation	Suicidal behavior
Psychomotor activity	0.61^*^	0.60^*^	0.51^*^
Verbal response	0.52^*^	0.51^*^	0.42^*^
Repetitive movements	0.39^*^	0.41^*^	0.41^*^
Artificial expressions	0.34^*^	0.34^*^	0.28^*^
Oppositivity	0.49^*^	0.47^*^	0.43^*^
Automatic obedience	0.37^*^	0.38^*^	0.28^*^
Automatisms	0.42^*^	0.41^*^	0.35^*^
Impulsivity	0.60^*^	0.56^*^	0.54^*^
Total score	0.62^*^	0.61^*^	0.52^*^

^*^Significant correlation for *p* < 0.05.

**Table 3:** Pearson’s correlations coefficients (*r*) among MOOD suicidality score and CS scores in the MDD group.

CS scores	Suicidality	Suicidality ideation	Suicidal behavior
Psychomotor activity	0.45^*^	0.43^*^	0.33^*^
Verbal response	0.47^*^	0.47^*^	0.31^*^
Repetitive movements	0.40^*^	0.39^*^	0.29^*^
Artificial expressions	0.38^*^	0.36^*^	0.31^*^
Oppositivity	0.50^*^	0.48^*^	0.38^*^
Automatic obedience	0.35^*^	0.38^*^	0.19^*^
Automatisms	0.32^*^	0.30^*^	0.26^*^
Impulsivity	0.46^*^	0.39^*^	0.44^*^
Total score	0.53^*^	0.51^*^	0.41^*^

^*^Significant correlation for *p* < 0.05.

**Table 4:** Pearson’s correlations coefficients (*r*) among MOOD suicidality score and CS scores in the BPD group.

CS scores	Suicidality	Suicidality ideation	Suicidal behavior
Psychomotor activity	0.51^*^	0.49^*^	0.43^*^
Verbal response	0.41^*^	0.38^*^	0.39^*^
Repetitive movements	0.24^*^	0.24^*^	0.18
Artificial expressions	0.08	0.11	0.01
Oppositivity	0.36^*^	0.33^*^	0.36^*^
Automatic obedience	0.33^*^	0.34^*^	0.25^*^
Automatisms	0.32^*^	0.32^*^	0.24^*^
Impulsivity	0.47^*^	0.46^*^	0.39^*^
Total score	0.49^*^	0.48^*^	0.41^*^

^*^Significant correlation for *p* < 0.05.

**Table 5:** Pearson’s correlations coefficients (*r*) among MOOD suicidality score and CS scores in the CTL group.

CS scores	Suicidality	Suicidality ideation	Suicidal behavior
Psychomotor activity	0.43^*^	0.43^*^	0.31^*^
Verbal response	0.26^*^	0.27^*^	0.15
Repetitive movements	0.28^*^	0.27^*^	0.24^*^
Artificial expressions	0.18^*^	0.18^*^	0.14
Oppositivity	0.27^*^	0.27^*^	0.22^*^
Automatic obedience	0.26^*^	0.25^*^	0.22^*^
Automatisms	0.27^*^	0.29^*^	0.16
Impulsivity	0.41^*^	0.40^*^	0.32^*^
Total score	0.41^*^	0.41^*^	0.30^*^

^*^Significant correlation for *p* < 0.05.

**Figure 1:**
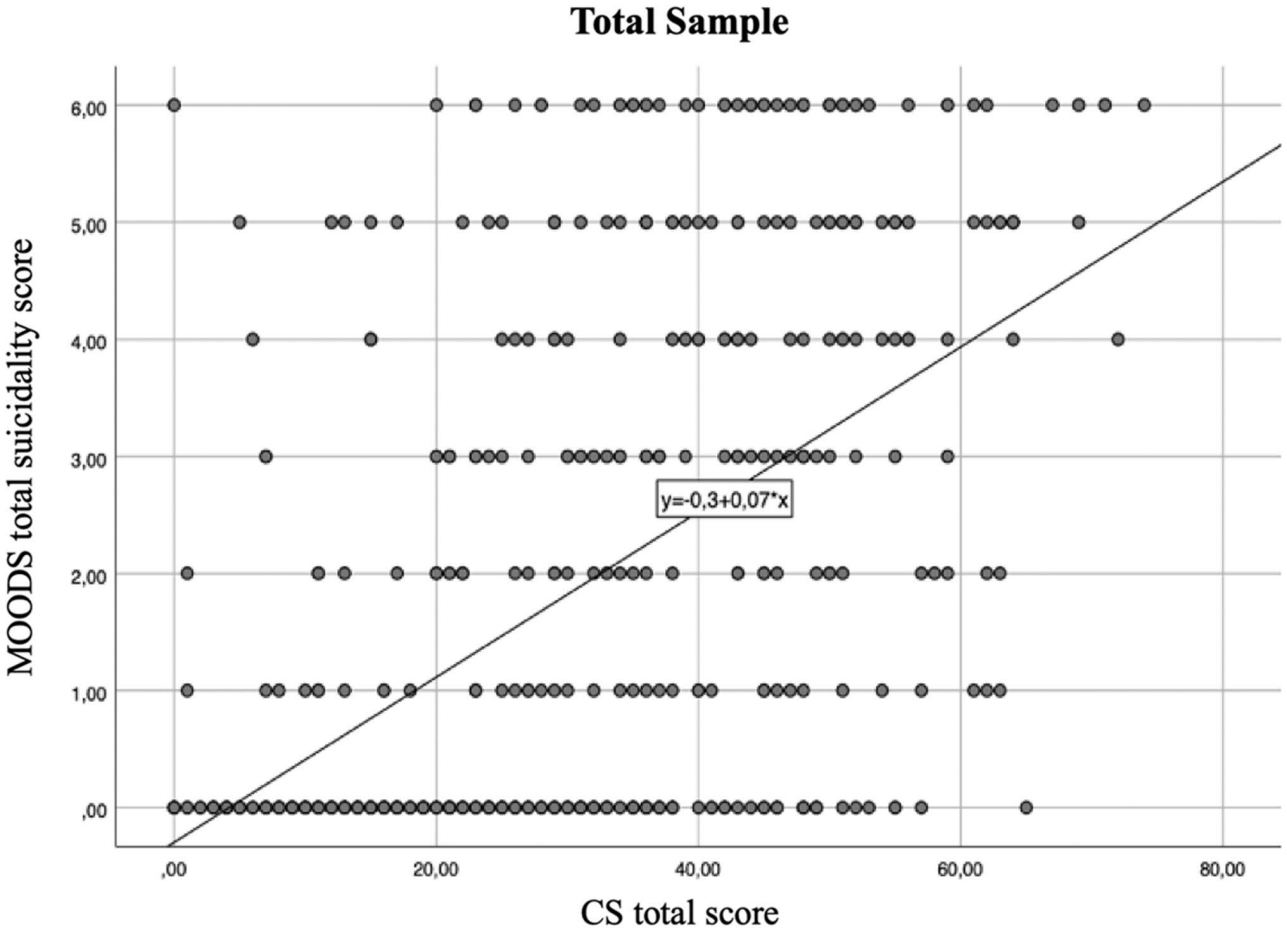
Regression line between suicidality score and catatonia spectrum (CS) total score in the total sample linear *R*^2^ = 0.408.

**Figure 2:**
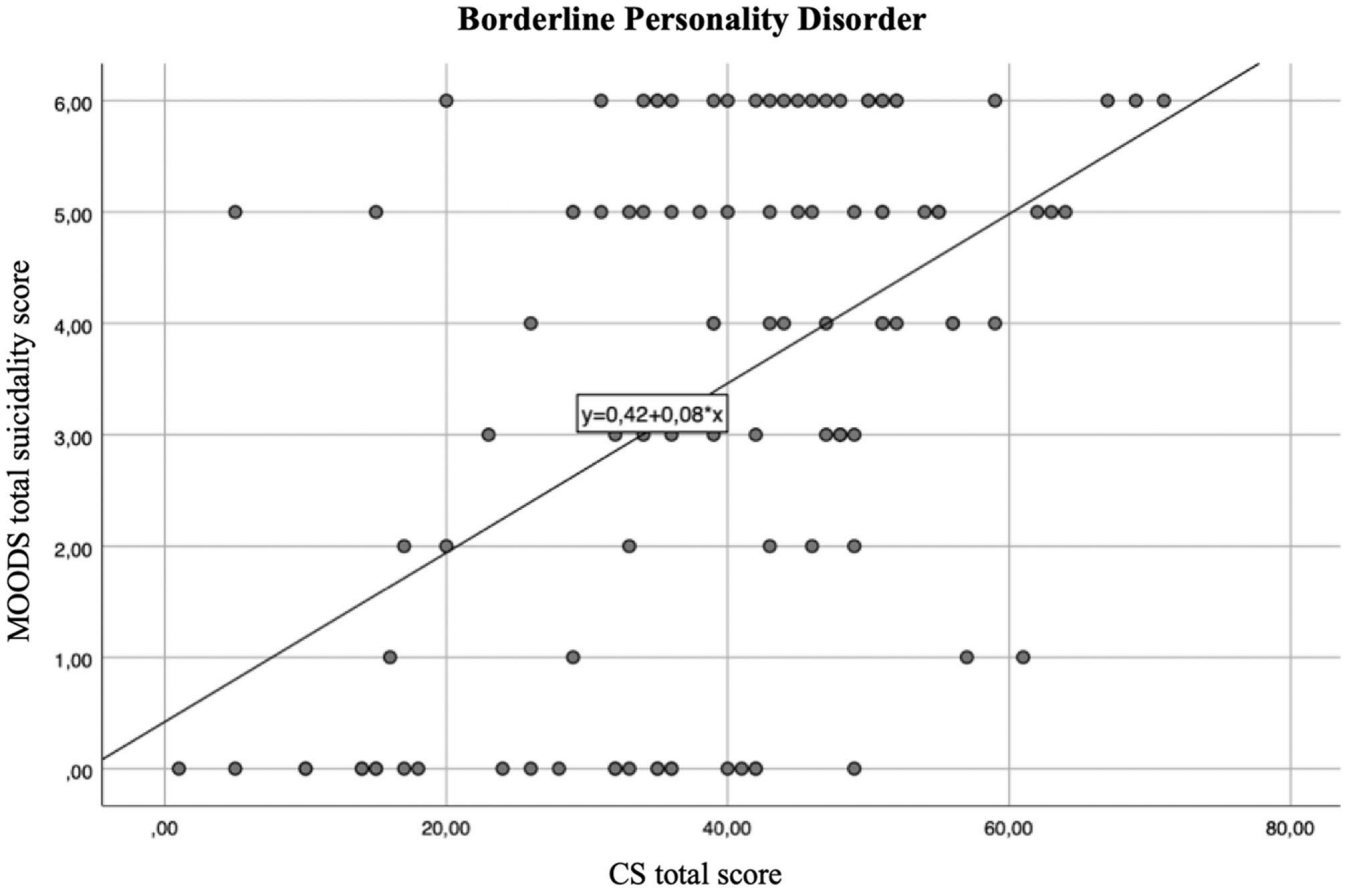
Regression line between suicidality score and CS total score in the BPD group linear *R*^2^ = 0.241.

The regression line showed that for every positive item of the CS the suicidality score increases of 0.08 in the total sample (Figure 1), and of 0.08 and 0.07 in the BPD and MDD groups, respectively (Figures 2 3). Finally, results from linear regression analysis showed that CS total score was a significant predictor of a higher suicidality score, as well as CS *psychomotor activity* (beta = 0.35; t = 5.57; *p* < 0.001), *verbal response* (beta = 0.11; *t* = 1. 99; *p* = 0.047) and *Impulsivity* (beta = 0.38; *t* = 6.32; *p* < 0.001) domain scores, while *automatism* was a negative predictor (beta = −0.14; *t* = −2.35; *p* = 0.02) (Table 6); meanwhile results from logistic regression analysis reported that the CS total score and CS *psychomotor activity*, *verbal response* and *impulsivity* (Table 7) domain scores were significant positive predictors of the presence of at least one positive response in the items evaluating the presence of suicidal thoughts whereas only *psychomotor activity* and *impulsivity* domain scores were significant positive predictors of the presence of at least one positive response in the items evaluating the suicidal behaviors (Table 8).

**Figure 3:**
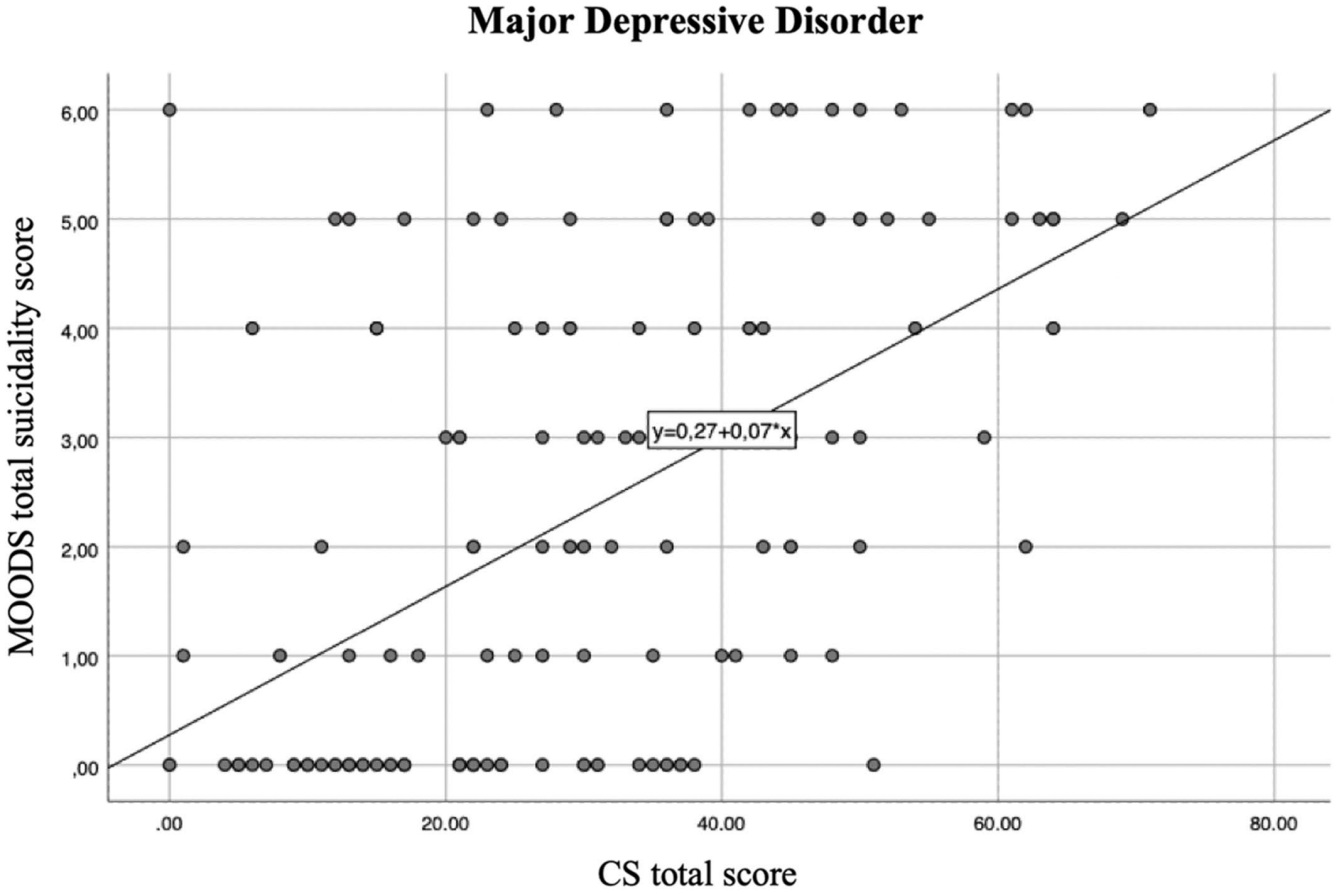
Regression line between suicidality score and CS total score in the MDD group linear *R*^2^ = 0.281.

**Table 6:** Linear regression analysis with MOODS suicidality score as a dependent variable and CS domains as independent variables in the total sample.

	*b* (SE)	BETA	*t*	*p*	CI 95%	
					Lower bound	Upper bound
Constant	−0.489 (0.171)	–	−2.856	0.005	−0.826	−0.152
CS total score	0.083 (0.005)	0.620	15.930	<0.001	0.0.73	0.093
Constant	−0.596 (0.184)	–	−3.097	0.002	−0.930	−0.208
Psychomotor act.	0.185 (0.033)	0.349	5.570	<0.001	0.120	0.250
Verbal response	0.097 (0.049)	0.115	1.988	0.047	0.001	0.193
Automatism	−0.111 (0.47)	−0.139	−2.355	0.19	−0.203	−0.18
Impulsivity	0.231 (0.037)	0.377	6.316	<0.001	0.159	0.303

**Table 7:** Logistic regression analysis with the presence of at least one positive MOOD Suicidal ideation item as a dependent variable and CS domains as independent variables in the total sample.

	*b* (SE)	*p*	Exp (*B*)	CI 95%	
				Lower bound	Upper bound
Constant	−3.023 (0.313)	<0.001	0.049		
CS total score	0.109 (0.011)	<0.001	1.115	1.092	1.139
Cox & Snell *R* square = 0.356; Nagelkerke *R* square = 0.475					
Constant	−3.3478 (0.387)	<0.001	0.031		
Psychomotor activity	0.255 (0.051)	<0.001	1.291	1.168	1.427
Verbal response	0.238 (0.075)	0.001	1.269	1.096	1.469
Impulsivity	0.207 (0.058)	<0.001	1.230	1.097	1.378

Cox & Snell *R* square = 0.400; Nagelkerke *R* square = 0.534.

**Table 8:** Logistic regression analysis with the presence of at least one positive MOOD Suicidal behavior item as a dependent variable and CS domains as independent variables in the total sample.

	*b* (SE)	*p*	Exp (*B*)	CI 95%	
				Lower bound	Upper bound
Constant	−3.592 (0.344)	<0.001	0.028		
CS total score	0.87 (0.009)	<0.001	1.090	1.071	1.111
Cox & Snell *R* square = 0.267; Nagelkerke *R* square = 0.378					
Constant	−3.874 (0.413)	<0.001	0.021		
Psychomotor activity	0.188 (0.054)	0.001	1.206	1.085	1.342
Impulsivity	0.289 (0.057)	<0.001	1.335	1.194	1.493

Cox & Snell *R* square = 0.321; Nagelkerke *R* square = 0.455.

## 4. Discussion

The aim of this study was to evaluate the presence and the prevalence of catatonic symptoms and how they may shape and correlate with suicidal risk in a sample of individuals with MDD or BPD, investigating also if they could represent statistically predictive factors of suicidality. Embracing the modern definition of the catatonic clinical picture, brought by the DSM-5-TR, which seems to be receptive of a less strictly categorical conceptualization and open to an expansion of its boundaries towards a dimensional approach, the Catatonia Spectrum model aims to evaluate the presence and correlates of catatonic sub-threshold and full-threshold symptoms in different clinical conditions (59). The “catatonic spectrum” comprehends a wide range of manifestations with various degrees of severity, consistent with the conceptualization of catatonia as a trans-nosographic specifier, potentially associated with every mental disorder (58).

As expected, we found that both clinical groups showed greater suicidality scores than controls. Noticeably, BPD patients showed increased suicidal behaviors than MDD ones, supporting the strong link between BPD and risk of suicidal attempts (60, 61).

In addition, we found strong correlation between the presence of catatonic symptoms, as evaluated by the CS, and suicidality, as measured by the MOODS suicidality items, in the total sample and in all groups. Increasing evidence is stressing a higher prevalence of catatonic manifestations in several mental disorders: in particular, about 10% of patients with severe acute psychiatric illness are reported to exhibit motor signs (including mutism, negativism, rigidity, stereotypy, etc.) that are identified as catatonia (62), and most of the cases involve individuals with depressive or bipolar disorders (2). In particular, a recent study reported high levels of catatonic manifestations in the patients affected by BPD (19), a severe mental disorder strongly correlated with suicidal behaviors (63, 64). Both disorders, as well as catatonia of psychiatric origin, are well known for the increased risk of suicidality, and our results seem to be in line with these evidences (20, 21).

It should be noted that previous studies also highlighted in both BPD and MDD populations a significant prevalence of autistic traits, even though they may differ in terms of quality and/or quantity, among individuals with mood disorders and BPD (49, 52, 65, 66), which also seem to increase the suicidal risk in both the categories of subjects (51).

Moreover, in the past decades, particular attention has been dedicated to the relationship between catatonia and ASD (58), thanks to the many shared clinical features shared by the two disorders, many authors have indicated that catatonia may slowly develop over the course of ASD (67), supporting the hypothesis of the presence of a psychopathological continuum starting from a neuroatypical vulnerability, and progressing and culminating in the manifestations of the catatonic spectrum, trespassing affective and personality disorders (62, 68). Interestingly, the presence of a positive correlation between suicidality score and most of the CS domains also in the CTL group support the hypothesis of a connection between catatonic traits and suicidality even in a subthreshold context of non full-blown psychopathologies.

Our findings also highlighted the possible presence of a specific pattern of features that seems to be predictive of suicidality. Considering the correlation analysis, in the MDD group all CS domains and the total score were significantly and positively correlated with suicidality scores, suicidal ideation and suicidal behavior, globally supporting the strong link between catatonic spectrum and suicidality (20, 21). Similarly, in the BPD group significant correlations were reported between suicidality total score, suicidal ideation, suicidal behavior and all CS domains, with the exception of CS *artificial expressions* domain and, only for suicidal behavior, of CS *repetitive movement domain.*

However, results from the regression analysis revealed that, besides the CS total score, specific catatonic symptoms seem to be statistically predictive of suicidality in the sample. In particular when considering the CS mean score as the dependent variable, the predictive factor for suicidality were the CS *psychomotor activity*, *verbal response* and *impulsivity* were identified as positive predictors, while *automatism* as a negative predictor. Similarly, CS *psychomotor activity*, *verbal response* and *impulsivity* domains emerged as positive predictors of suicidal ideation, while only CS *psychomotor activity*, and *impulsivity* were highlighted as significant predictors of suicidal behavior.

The domains *psychomotor activity* (stupor) and *impulsivity* represent the two most severe and extreme clinical presentation of catatonia. Noticeably, one of the factors which was already hypothesized to be at the basis of catatonic episodes is the feeling urged towards two opposite actions at the same time: an ambi-tendence. Clinically, catatonia is not just petrifying paralysis, but a complex condition in which psychomotor activity is reduced, excessive or abnormal, in a so-called psychomotor alternation. Following the theory proposed by Fink (69) for which catatonia is often linked to and can be driven by fear (70), and that the signs of catatonia may be adaptations to persistent fear, similar to a tonic immobilization, the manifestation of stupor and impulsivity could easily be compared to the animal behavior in front of danger where the freeze reaction is the catatonic extreme form of total psychomotor block and the flight one would correspond to agitation and impulsive behaviors (71). In this view, the two extreme and opposite manifestations are the main indicators of the gravity of the catatonic manifestation, explaining also the raised suicidal risk and the specific link with suicidal behavior. This assumption in also supported by the fact that the sedation derived from the benzodiazepines can afford relief to for the fear that occupies the mind of the catatonic patient (69).

Suicidality total score and suicidal ideation was instead found to be associated also with the CS *verbal response* domain. The presence of deficits in the verbal and non-verbal communication has often been reported to be linked to suicidality, especially in ASD patients, where they could lead to a higher risk of isolation and depressive symptoms (57, 72, 73). This correlation is also in line with previous studies that highlighted how a greater awareness of the self-difficulties in communicating with others can lead to a greater distress (74, 75).

On the other side, the *artificial expressions* domain portrays the clinical symptom of catatonic mannerism. Noticeably, this dimension seemed to negative predict suicidality scores according to our results. Although the available literature lacks to report a hypothetic link between mannerism and suicidality or its protective effect, we can assume that mannerism manifestations are partially similar to camouflaging, featuring exaggerated, abnormal expression of common gestures and behaviors (76). Despite previous research highlighted instead a possible positive link between camouflaging behavior and suicidality (77), the recent literature has also focused on camouflaging as a strategy adopted to cope with social environment, in order to reach a greater integration between verbal and nonverbal behaviors, a greater reciprocity in conversations or relationships, and minimizing responses toward sensory over stimulations (74, 78, 79). In this view, camouflaging and mannerism, for some individuals might result in a better adjustment (75, 80).

This study should be considered in light of some obvious limitations. Firstly, the cross-sectional design of the study prevented us to evaluate possible temporal relationships between the considered variables. Moreover, we recognize that the optimal way to investigate suicidal ideation and behaviors should be *via* clinical evaluation, and that the use of self-report instruments may have led to over- or underestimations of symptoms by the subjects, and to consequent biases in our results. Thirdly, this study does not report the clinical characteristics nor other component of MOODS-SR (such as energy, cognition and rhythmicity) for they are analyzed elsewhere. Furthermore, the presence of substance misuse was not investigated, nor the use pharmacological therapies whose current use or withdrawal could have cause catatonic manifestations. Additionally, no blinding procedures were used for the study and this possibly could have implied a bias in the interpretation of the results. Finally, the relatively small sample size limits the extensibility of the present work. Further study in wider sample and with a longitudinal design are needed for clarifying the relationship between catatonic spectrum and suicidality in different clinical conditions.

## 5. Conclusion

In the context of the above limitations, results from this study seem to globally highlight that higher catatonic manifestations are linked to higher suicidal tendencies, confirming the high risk of suicide for patients falling under the umbrella of the “catatonic spectrum,” conceptualized as a wider range of manifestations with various degrees of severity. Further studies should address the presence of specific patterns of catatonic symptoms and traits in different mental disorders, such as affective and personality ones, and how they might affect psychopathological trajectories and clinical outcomes.

## Data availability statement

The original contributions presented in the study are included in the article/supplementary material, further inquiries can be directed to the corresponding author.

## Ethics statement

The studies involving human participants were reviewed and approved by Ethics Committee of the Azienda Ospedaliero-Universitaria of Pisa. The patients/participants provided their written informed consent to participate in this study.

## Author contributions

LD conceived the work. LD, BN, CB, DG, FB, GA, SB, GM, ML, IB, NB, MG, GD, MN, AR, MMu, MP, PP, AV, CC, IC, BC, and MMa collected the data processed in the study. GM did statistical analysis. BN and LD drafted the manuscript and revised the work. All authors contributed to the article and approved the submitted version.

## Conflict of interest

The authors declare that the research was conducted in the absence of any commercial or financial relationships that could be construed as a potential conflict of interest.

## Publisher’s note

All claims expressed in this article are solely those of the authors and do not necessarily represent those of their affiliated organizations, or those of the publisher, the editors and the reviewers. Any product that may be evaluated in this article, or claim that may be made by its manufacturer, is not guaranteed or endorsed by the publisher.
